# Delgocitinib cream for the treatment of refractory vulvar lichen sclerosus

**DOI:** 10.1016/j.jdcr.2026.05.051

**Published:** 2026-05-27

**Authors:** Paige L. McKenzie, Ana Preda-Naumescu, Christopher G. Bunick

**Affiliations:** a Department of Dermatology, Yale School of Medicine, New Haven, Connecticut; b Program in Translational Biomedicine, Yale School of Medicine, New Haven, Connecticut; c Institute for Global Health, Yale School of Medicine, New Haven, Connecticut

**Keywords:** chronic inflammatory dermatosis, cutaneous atrophy, fibrosis, genital skin disease, itch, topical JAK inhibitor therapy

## Introduction

Lichen sclerosus (LS) is a chronic inflammatory skin disease commonly affecting the genital and perianal regions. In women, vulvar LS causes severe and persistent pruritus and can lead to progressive scar-like atrophy and functional impairment, and increased risk of vulvar malignancy.^1^^,^^2^ Extragenital involvement, although rare, may occur. Ultrapotent topical corticosteroids are the recommended first-line therapy, with topical calcineurin inhibitors commonly used as second-line agents.^3^ Some patients, however, have persistent symptoms or adverse effects with long-term use of these therapies. We report a case of refractory vulvar LS with severe pruritus that responded to treatment with delgocitinib cream, a topical Janus kinase inhibitor not previously described for this indication.

## Case report

**Fig 1:**
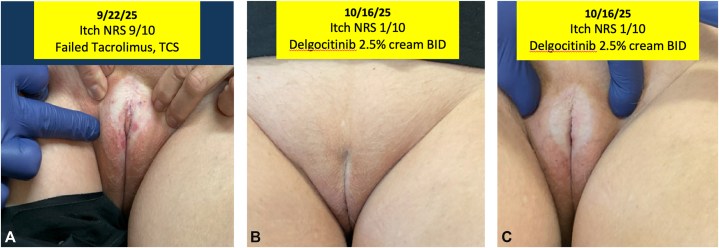
**A,** A 74-year-old woman with history of vulvar lichen sclerosus and chronic pruritus refractory to topical clobetasol and tacrolimus × 12 m; **(B, C)** response to delgocitinib (Anzupgo) 2.5% cream twice daily × 1 m. *NRS*, Numeric rating scale.

A 74-year-old White woman presented with a 3-year long history of genital pruritus. Her past medical history included chronic lymphocytic leukemia, basal and squamous cell carcinomas, gastroesophageal reflux disease, and fibromyalgia. On exam, the patient had sharply circumscribed, atrophic white plaques with overlying excoriations surrounding the clitoris and labia minora with extension to the anterior labia majora bilaterally. A clinical diagnosis of vulvar LS was made. The patient was initially treated with tacrolimus 0.1% ointment twice daily for 4 weeks without improvement. She was then instructed to start clobetasol propionate 0.05% cream twice daily for 2 weeks alternating with tacrolimus 0.1% ointment twice daily for 2 weeks each month. She trialed this regimen for approximately 12 months without significant improvement in genital pruritus (Fig 1, *A*). On initial follow-up, her worst-itch numeric rating score (WI-NRS) was 9 of 10. Given that her disease was refractory to standard therapies, the patient was prescribed delgocitinib 2.5% cream for use twice daily. Within 1 month of delgocitinib use, her WI-NRS decreased from 9 to 1 of 10, and the LS plaque decreased in size by approximately 1 cm at the superior aspect and was without overlying erosions (Fig 1, *B* and *C*). A vaginal superficial aerobic culture was obtained at this time from untreated, eroded areas on the occluded labial surfaces, which grew 4+ *Pseudomonas aeruginosa* and 2+ beta-hemolytic streptococcus group B; the patient was treated with oral ciprofloxacin 500 mg twice daily for 5 days and cephalexin 500 mg three times daily for 10 days.

## Discussion

This patient with refractory vulvar LS experienced marked improvement in severe pruritus and clinical disease activity following treatment with topical delgocitinib, a pan–Janus kinase (JAK) inhibitor. This response is notable given her lack of meaningful improvement after prolonged use of first-line ultrapotent topical corticosteroids and topical calcineurin inhibitors. To our knowledge, this is the first reported case describing delgocitinib cream for vulvar LS.

LS is a chronic inflammatory dermatosis with a complex and incompletely understood pathogenesis. Emerging evidence suggests cytokine-mediated immune dysregulation, including signaling through the JAK–signal transducer and activator of transcription (STAT) pathway, may contribute to disease activity.^4^ Elevated expression of proinflammatory cytokines, including interferon-γ and interleukin-6, is documented in patients with LS.^4^ Interferons and interleukins are among the factors that can activate JAK/STAT signaling, providing a mechanistic rationale for targeting downstream JAK signaling.^4^ When topical steroids or calcineurin inhibitors fail in LS,^1^^,^^5^ off-label use of acitretin or methotrexate has historically been trialed, though long-term control of disease with these oral agents remains unsatisfactory.^5^ While systemic JAK inhibitors have shown efficacy in a variety of inflammatory and autoimmune skin diseases, their use in LS has been limited. More recently, several JAK inhibitors have been explored for the treatment of LS.^5^ Baricitinib, an oral JAK1/2 inhibitor approved for the treatment of alopecia areata in the United States, has shown favorable data in both a single-arm prospective study and case reports for the treatment of genital and extragenital LS.^6^ Similarly, abrocitinib, an oral JAK 1 inhibitor approved for management of atopic dermatitis (AD), has shown efficacy in both vulvar LS and the male penile equivalent, balanitis xerotica obliterans.^7^^,^^8^ Finally, tofacitinib, an inhibitor of JAK1/3, has shown benefit in knee-joint contractures and thickening of the skin in a patient with bullous LS-generalized morphea overlap syndrome.^9^

While systemic JAKs are increasingly recognized as potential treatment options for genital and extragenital LS, the literature surrounding the use of topical JAK inhibitors is largely limited to case reports and case series. Topical ruxolitinib 1.5% cream, a JAK 1/2 inhibitor currently approved for AD and vitiligo, has shown promise in pediatric cases of genital LS, and adult cases of extragenital or generalized LS.^10^^,^^11^

Delgocitinib is approved for the treatment of chronic hand eczema. In our patient, delgocitinib rapidly reduced pruritus, as reflected by a decrease in WI-NRS from 9 to 1 within 1 month, along with visible clinical improvement of the vulva. Given the severity and chronicity of this patient’s symptoms, and failure of response to standard therapies, this case supports a role for topical pan-JAK inhibition in refractory LS. Notably, while delgocitinib led to marked improvement in pruritus and inflammatory disease activity, there was no appreciable reversal of established sclerotic or atrophic changes, suggesting JAK inhibition may primarily target active inflammation rather than preexisting fibrosis.

It is worth noting that this patient’s course was complicated by superimposed bacterial colonization, which was treated with systemic antibiotics. However, the temporal association between initiation of delgocitinib and symptomatic improvement, as well as the degree of itch reduction prior to antimicrobial therapy, supports a primary therapeutic effect of JAK inhibition rather than resolution of infection alone. While both topical corticosteroids and delgocitinib are reported to increase rates of localized cutaneous infections, topical delgocitinib carries significantly lower risk of other local adverse effects, particularly skin atrophy, telangiectasia, striae, and hypopigmentation, compared to topical corticosteroids. Data from patients with AD on the trunk and extremities showed that switching from topical corticosteroids to delgocitinib resulted in >20% reduction in severity of skin atrophy and telangiectasia.^12^

As the number of patients with LS who are refractory to or intolerant of long-term topical corticosteroid therapy continues to grow, there is a clear need for alternative topical treatments. This case highlights topical delgocitinib as a promising therapeutic option and supports further investigation into the role of JAK–STAT signaling in LS pathophysiology. Larger studies are needed to better define efficacy, optimal treatment duration, and long-term safety. Until such data are available, this case adds to the emerging body of evidence supporting targeted topical immune modulation as a viable strategy in difficult-to-treat vulvar LS.

## Conflicts of interest

Dr Bunick has served as an investigator and consultant for LEO Pharma. Drs McKenzie and Preda-Naumescu have no conflicts of interest to declare.
